# Explainable temporal machine learning of multimorbidity trajectories after acute myocardial infarction: complementing clinical risk scores with mechanistic phenotypes

**DOI:** 10.1093/jamia/ocag135

**Published:** 2026-08-06

**Authors:** Anthony Onoja, Kris Elomaa, Anthony D Whetton, Nophar Geifman

**Affiliations:** School of Health Sciences, Faculty of Health and Medical Sciences, University of Surrey, Guildford, GU2 7XH, United Kingdom; School of Health Sciences, Faculty of Health and Medical Sciences, University of Surrey, Guildford, GU2 7XH, United Kingdom; School of Biosciences and Veterinary Health Innovation Engine, School of Veterinary Medicine, University of Surrey, Guildford, GU2 7XH, United Kingdom; School of Health Sciences, Faculty of Health and Medical Sciences, University of Surrey, Guildford, GU2 7XH, United Kingdom

**Keywords:** multimorbidity, myocardial infarction, machine learning, cluster analysis, risk stratification

## Abstract

**Objective:**

This study aimed to identify trajectories of multimorbidity following acute myocardial infarction (AMI), using explainable temporal machine-learning methods, and assess their clinical, prognostic, and biological significance.

**Materials and Methods:**

Dynamic Time Warping k-means clustering was applied to post-AMI diagnostic sequences from 12 701 UK-Biobank participants. Latent Dirichlet Allocation characterised cluster themes. Multiclass classifiers (CatBoost, XGBoost, random forest, logistic regression) trained on pre-AMI diagnoses and demographics predicted trajectory membership, with SHAP interpretability. SMART scores and Cox models evaluated 5-year mortality; Phenotype-Wide Association (PheWAS) and Reactome pathway enrichment were used to identify associated biological mechanisms.

**Results:**

Three trajectories of multimorbidity were identified: acute cardiorenal-respiratory with metabolic disease (ACUTE-CARD; 63.4%), cardiometabolic disease with arrhythmic-ischemic burden (CARDIOMIX; 13.5%), and smoking-related multisystem multimorbidity (SMO-CARD; 23.1%). XGBoost achieved the highest discrimination (AUC-ROC 0.906; 95% CI, 0.895-0.916), with CatBoost showing comparable performance (AUC-ROC 0.900; 95% CI, 0.889-0.910). SMO-CARD had the highest 5-year mortality (43.9%). The established SMART cardiovascular risk score remained the dominant predictor of mortality while the identified trajectories provided complementary prognostic signals; these associations were weaker after full adjustment for potential confounders with each profile displaying distinct genetic and pathway signatures.

**Discussion:**

The SMART score outperformed in capturing mortality risk, whereas the trajectories complement it by revealing nuanced clustering of risk factors across organ systems and in identifying trajectory-specific intervention priorities.

**Conclusion:**

Explainable temporal modeling of EHR data reveals clinically interpretable, biologically grounded multimorbidity trajectories after AMI that complement established risk scores and provide a reproducible approach to mechanistic phenotyping and precision care.

## Introduction

Multimorbidity, the coexistence of other conditions, is the dominant clinical reality for patients with cardiovascular disease. Individuals who experience an acute myocardial infarction (AMI) commonly live with chronic conditions across metabolic, renal, respiratory, vascular, neurological, and musculoskeletal systems.^1–5^ These co-existing diseases evolve over time and shape functional status, healthcare utilisation, and survival.^6^^,^^7^ Yet most electronic health record (EHR)-based tools treat comorbidity as a static construct, summarising diagnoses into counts or composite indices.^8–11^ Such measures are useful for capturing overall burden but ignore the temporal evolution of disease and offer limited insight into which biological systems drive risk in each patient.^12^^,^^13^

Cardiovascular risk scores such as the Second Manifestations of Arterial Disease (SMART) score provide robust predictions of 10-year and lifetime recurrent vascular risk by combining traditional factors including age, sex, blood pressure, smoking, and lipids.^14^ In practice they function as highly optimised mortality calculators. However, they do not distinguish whether a high-risk estimate reflects advanced frailty, predominantly cardiometabolic disease, or other multimorbid patterns, and they offer little guidance on how to tailor interventions beyond guideline-directed therapy.^11^^,^^15–18^ Within medical informatics there is increasing interest in modeling longitudinal trajectories of diagnoses recorded in EHRs.^9^^,^^12^^,^^13^ Temporal methods, including Dynamic Time Warping, enable comparison of patient-level sequences even when disease progression occurs at different speeds, and clustering can reveal coherent multimorbidity patterns that are not apparent in cross-sectional data.^19^ Adoption has been limited, however, by concerns about model opacity and by the lack of large-scale studies that link temporal clusters to prognosis and underlying biology.^20–23^ Explainable artificial intelligence techniques such as Shapley Additive Explanations (SHAP) and topic modeling can mitigate this by providing transparent, clinically interpretable summaries of complex models.^24–26^

In this study, we apply and evaluate explainable temporal modeling methods for multimorbidity using linked EHR data from UK Biobank. Using patients with an incident AMI as a case study, we represent post-infarction diagnoses as age-indexed time series and apply Dynamic Time Warping–based clustering to identify groups of patients who share similar trajectories of disease accumulation over 5 years. We characterise these clusters with topic modeling, examine whether cluster membership can be predicted from pre-AMI diagnoses and demographic characteristics using supervised learning and SHAP explanations, and assess their association with 5-year all-cause mortality alongside the SMART risk score. Finally, we explore whether the derived trajectory-based profiles correspond to distinct genetic associations and molecular pathways. We hypothesised that temporal clustering would yield clinically interpretable and biologically distinct trajectories, that these trajectories would be predictable from pre-AMI information, and that they would provide complementary prognostic and mechanistic insight when combined with traditional risk stratification tools (see Figure  1).^10^^,^^27–29^

**Figure 1.**
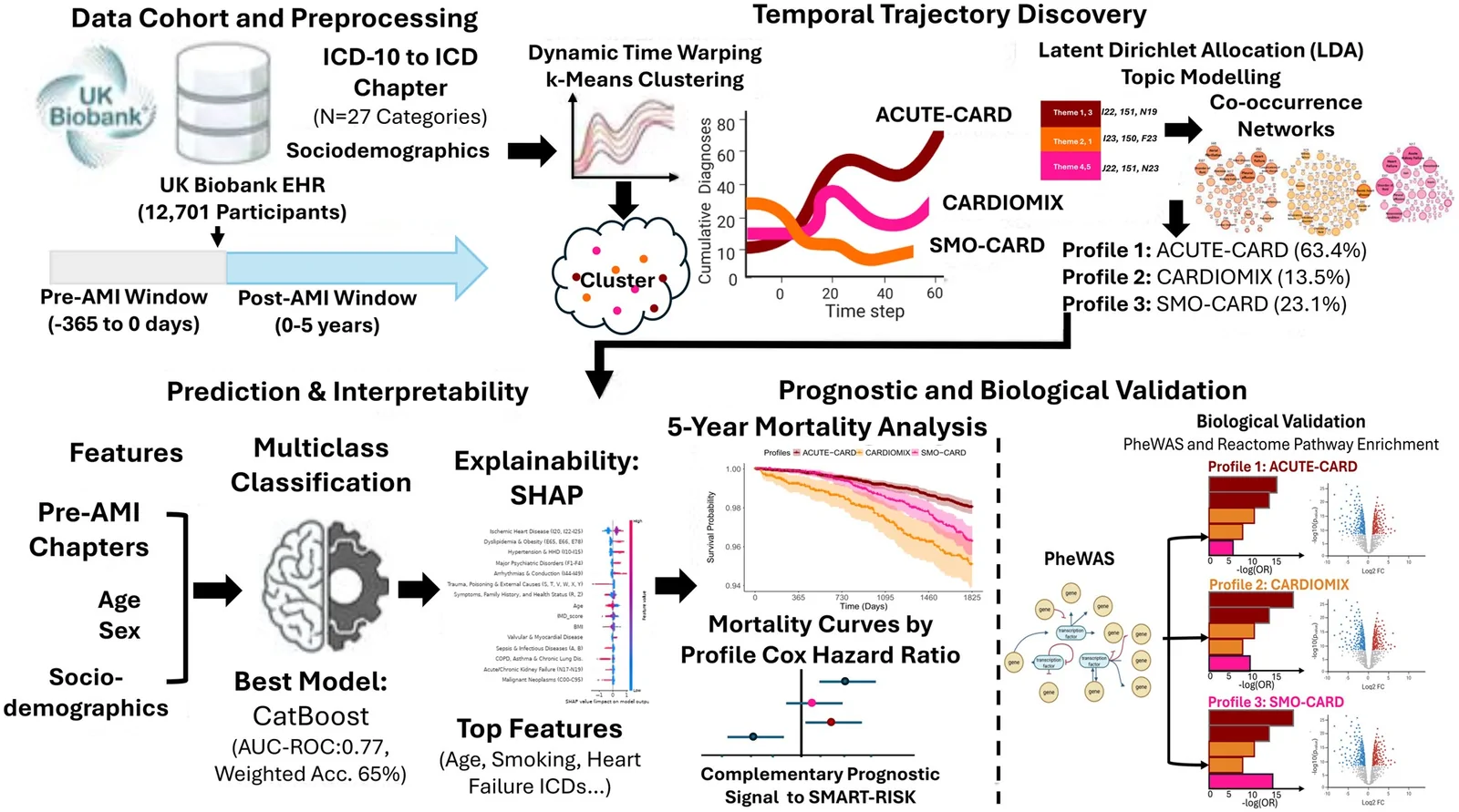
Schematic of the analytical workflow and computational pipeline: (1) data collection and structuring of ICD‑10 age‑at‑diagnosis matrix, (2) temporal sequence construction and DTW clustering, (3) creation of pre‑AMI feature matrix and train/test split, (4) supervised learning and SHAP interpretation, (5) network analysis and PheWAS mapping with pathway enrichment, and (6) survival modeling with SMART risk and covariates.

## Methods

### Study design and population

We conducted a retrospective cohort study using UK Biobank data (application 83 988), a population-based resource of ∼500 000 participants aged 40-69 years recruited 2006 and 2010, with longitudinal linkage to hospital episode statistics, death registries, and primary care records.^27^

Participants were eligible if they had an incident AMI (ICD-10 I21) with at least 5 years of follow-up or death within that window, no missing demographic data, and no implausible dates. In total, 12 701 participants met inclusion criteria.

### Diagnostic data processing

Hospital inpatient diagnoses (ICD-10)^30–32^ were filtered to remove pregnancy, perinatal, injury, nonspecific symptom, and transient infection codes. ICD-10 codes with fewer than 5 occurrences across the cohort and codes appearing fewer than 5 times were removed. Remaining codes were truncated to 3 characters, yielding 1008 distinct codes. Diagnoses were split into a pre-AMI window (365 days to 1 day before AMI) and a post-AMI window (0-5 years after AMI).

### Temporal trajectory representation and DTW clustering

Post-AMI diagnoses were encoded as age-indexed binary matrices: rows = ICD-10 codes; columns = integer ages from AMI to 5 years later; entries = 1 at first occurrence, 0 otherwise. Zero entries explicitly denote diagnostic stability, preserving temporal gaps. Ages were standardised using mean-variance transformation. Age-indexing was chosen over time-since-event to explicitly capture age-dependent disease susceptibility and progression; biological age is a primary driver of multimorbidity patterns, and aligning trajectories by age enables identification of age-stratified risk profiles that are clinically meaningful and prognostically relevant. While time-since-AMI may capture early vs late complications, age-indexing reflects the cumulative biological context in which these complications arise. DTW k-means clustering (tslearn v0.6.3) was applied; cluster number *k* = 3 was selected using elbow, silhouette, and Davies-Bouldin criteria over *k* = 2-9.

### Cluster characterisation

LDA (scikit-learn v1.3.2; 2 topics per cluster) was applied to post-AMI diagnosis vectors within each cluster. While LDA conventionally operates on word-frequency counts, its probabilistic framework is directly applicable to binary diagnostic data: each patient’s diagnosis record serves as a “document,” each ICD-10 code as a “word,” and clinical phenotypes as “topics.”^33^^,^^34^ LDA identifies co-occurring diagnostic patterns (topic-word distributions) even when code presence is binary, capturing systematic disease co-occurrences rather than word frequencies. Applying LDA independently within each DTW cluster ensures that the identified topics are specific to each trajectory sub-population, preserving within-cluster clinical coherence. Top codes were grouped into clinical categories by 2 independent clinicians and used to assign cluster labels. Inter-rater reliability was assessed using Cohen’s Kappa, yielding *kappa* = 0.939 (95% agreement across 60 evaluated diagnostic codes, with 4 minor category disagreements), indicating near-perfect agreement between reviewers. Disease co-occurrence networks were constructed from Pearson correlations between binary code indicators; edges above the 95th percentile were retained and visualised in Cytoscape v3.10.

### Supervised prediction and SHAP

Pre-AMI diagnoses from the primary window (365 to 1 day before AMI) were mapped to 27 ICD-10-based clinical categories; standardised counts were combined with age, sex, BMI, and Index of Multiple Deprivation (IMD) score. Four classifiers were evaluated (logistic regression, random forest, XGBoost, CatBoost) with grid search hyperparameter tuning (3-fold CV, weighted *F*1) and class weights inversely proportional to cluster frequency. The dataset was split 80/20 stratified by cluster. SHAP values explained per-feature contributions to each class prediction for the best model. As a sensitivity analysis to address potential information leakage, all models were additionally trained and evaluated on a restricted pre-AMI window of 365 to 7 days before AMI (excluding the final 7 days before the index event). Performance metrics (AUC-ROC and weighted *F*1) with 95% bootstrap confidence intervals (1000 resamples) were computed for all models under both the main and sensitivity windows and reported in Table S2.

### Survival analysis

SMART scores were computed at AMI incidence using UK Biobank baseline variables. Four sequential Cox proportional hazards models were fitted: (1) trajectory profile membership alone, (2) SMART score alone, (3) trajectory + SMART score, and (4) fully adjusted model including trajectory + SMART score, and adding age, sex, IMD, SBP, diabetes, prior CAD, cerebrovascular disease, and PAD. Discrimination was assessed by Harrell’s C-index; Kaplan-Meier curves were constructed for unadjusted analyses.

### Genetic and pathway analyses

PheWAS was performed by mapping cluster conditions to PheCodes^35^^,^^36^ and testing associations with UK Biobank genotyped SNPs, adjusting for age, sex, ancestry principal components, and genotyping array. Significant SNPs (FDR-corrected p< 0.05) were input to Reactome pathway enrichment (hypergeometric test, FDR correction). All analyses used Python 3.8.10. All analysis code (ICD-10 filtering, DTW clustering, supervised learning, SHAP, survival analysis, and PheWAS) is publicly available on GitHub at https://github.com/Donmaston09/AMI_multimorbidity_prediction (see Supplementary Materials for a full description of repository contents).

### Ethics approval

This study was conducted using the UK Biobank resource under approved application number 83 988. The UK Biobank study received ethical approval from the Northwest Multi‑centre Research Ethics Committee (REC reference: 06/MRE08/65), and all participants provided written informed consent at recruitment. All analyses in the present study were performed in accordance with the Declaration of Helsinki and relevant UK regulations governing the use of health‑related research data.

## Results

### Cohort characteristics

Among 12 701 UK Biobank participants with incident AMI and at least 5 years of follow-up or death, the median age at AMI was 69 years (IQR 63-74) and 30.9% were male. Multimorbidity was prevalent: participants had a median of 8 ICD-10 diagnoses post-AMI (range 0-66) and 4 in the pre-AMI year (range 0-47). The median SMART score for 10-year recurrent vascular risk was 11.8% (9.1-15.2) and annual all-cause mortality risk was 0.6% (0.2-0.9). Five-year all-cause mortality was 21.0% (2673 deaths). Decedents were older, had higher SMART scores, and carried greater multimorbidity burden than survivors (Table 1).

**Table 1.** Baseline characteristics of AMI participants stratified by multimorbidity profile.

Attributes	Whole AMI cohort (*n* = 12 701)	ACUTE-CARD (*n* = 8052)	CARDIOMIX (*n* = 1720)	SMO-CARD (*n* = 2929)	*P*-value
Sex (female) (%)	8778 (69.1)	5651 (70.2)	1202 (69.9)	1925 (65.7)	<.0001
Age (years)	62.0 (56.0, 66.0)	61.0 (55.0, 65.0)	61.0 (55.0, 65.0)	64.0 (60.0, 67.0)	<.0001
BMI	28.0 (25.4, 31.1)	27.8 (25.3, 30.7)	27.7 (25.2, 30.8)	28.6 (25.8, 32.2)	<.0001
IMD score	13.8 (7.5, 26.0)	13.5 (7.2, 24.8)	12.7 (6.9, 24.3)	15.6 (8.4, 29.8)	<.0001
Waist circumference	96.0 (88.0, 104.0)	95.0 (87.0, 103.0)	95.0 (87.0, 103.0)	98.0 (89.0, 107.0)	<.0001
Hip circumference	103.0 (98.0, 109.0)	103.0 (98.0, 108.0)	103.0 (98.0, 109.0)	104.0 (99.0, 111.0)	<.0001
Stroke (%)	821 (6.5)	357 (4.4)	90 (5.2)	374 (12.8)	<.0001
CVD death (%)	1224 (9.6)	546 (6.8)	203 (11.8)	475 (16.2)	<.0001
All-cause mortality (%)	2673 (21.0)	1034 (12.8)	354 (20.6)	1285 (43.9)	<.0001
Composite CVD (%)	435 (3.4)	200 (2.5)	70 (4.1)	165 (5.6)	<.0001
SMART score	11.8 (9.1, 15.2)	11.2 (8.9, 14.2)	11.2 (8.8, 14.8)	14.0 (10.4, 18.4)	<.0001
SMART REACH score CVD mortality	2.5 (2.0, 3.2)	2.4 (2.0, 3.1)	2.3 (1.8, 2.9)	2.8 (2.2, 3.8)	<.0001
SMART REACH score all-cause mortality	0.6 (0.2, 0.9)	0.5 (0.2, 0.8)	0.5 (0.2, 0.8)	0.8 (0.4, 1.2)	<.0001
SBP (mmHg)	144.0 (132.0, 156.0)	144.0 (132.0, 156.0)	142.0 (130.0, 156.0)	144.0 (132.0, 157.0)	.027
DBP (mmHg)	84.0 (77.0, 91.0)	85.0 (78.0, 92.0)	84.0 (77.0, 91.0)	82.0 (74.0, 90.0)	<.0001
Pulse rate (BPM)	69.0 (62.0, 78.0)	69.0 (62.0, 77.0)	68.0 (61.0, 77.0)	69.0 (61.0, 78.0)	.182
eGFR (mL/min/1.73 m^2^)	90.0 (80.0, 100.0)	91.0 (81.0, 101.0)	91.0 (81.0, 100.0)	85.0 (73.0, 96.0)	<.0001
Diabetes (%)	853 (6.7)	280 (3.5)	113 (6.6)	460 (15.7)	<.0001
CAD (%)	1235 (9.7)	318 (3.9)	254 (14.8)	663 (22.6)	<.0001
CD (%)	195 (1.5)	78 (1.0)	23 (1.3)	94 (3.2)	<.0001
AAA (%)	29 (0.2)	9 (0.1)	1 (0.1)	19 (0.6)	<.0001
PAD (%)	179 (1.4)	46 (0.6)	19 (1.1)	114 (3.9)	<.0001

The indices of IMD scores are based on the qualitative studies of deprived areas within the local councils of England, Wales, and Scotland published by the UK government at the time of the UK Biobank baseline visits. They are based on more factors than the traditionally used Townsend index and preferred by the UK Biobank.^43^ SMART risk score: estimates the 10-year risk of recurrent vascular events in participants with vascular disease. SMART (Second Manifestations of Arterial Disease Reduction of Atherothrombosis for Continued Health). SBP: Systolic blood pressure at baseline (mean of the 2 measurements, automated reading); Diastolic Blood Pressure: Diastolic blood pressure at baseline (mean of the 2 measurements, automated reading), Pulse rate at baseline (mean of the 2 measurements, automated reading, BPM), eGFR at baseline. Calculated using the UKB data for age, sex, serum creatinine, and cystatin C. Data in Table 1 are presented as number (percent) for categorical variables, median (25th, 75th percentile) for continuous variables.

Abbreviations: AAA, abdominal aortic aneurysm; ACUTE-CARD, acute cardio renal respiratory with chronic metabolic disease; BPM, beats per minute; CARDIOMIX, cardiometabolic disease with mixed arrhythmic-ischemic burden; CAD, coronary artery disease; CD, cerebrovascular disease; eGFR, estimated glomerular filtration rate; IMD, index of multiple deprivation; PAD, peripheral artery disease; SBP, systolic blood pressure; SMART, Second Manifestations of Arterial Disease; SMO-CARD, smoking-related cardiovascular disease and multimorbidity.

### Temporal multimorbidity trajectories

DTW k-means clustering identified 3 stable, clinically interpretable profiles (silhouette coefficient 0.45; Davies–Bouldin index 0.85; Figure 2). ACUTE-CARD (*n* = 8052; 63.4%) was characterised by early clustering cardiometabolic diagnoses—hypertension, dyslipidemia, type 2 diabetes, and ischemic heart disease with episodic arrhythmia and acute cardiorespiratory decompensation and had the lowest 5-year mortality. CARDIOMIX (*n* = 1720; 13.5%) showed steady accumulation of structural heart disease, atrial fibrillation, valvular disease, and cardiorenal metabolic dysfunction, with intermediate mortality. SMO-CARD (*n* = 2929; 23.1%) exhibited progressive multisystem disease spanning COPD, respiratory failure, musculoskeletal disorders, and frailty markers; participants were older, more socioeconomically deprived, and had the highest 5-year mortality (43.9%).

**Figure 2.**
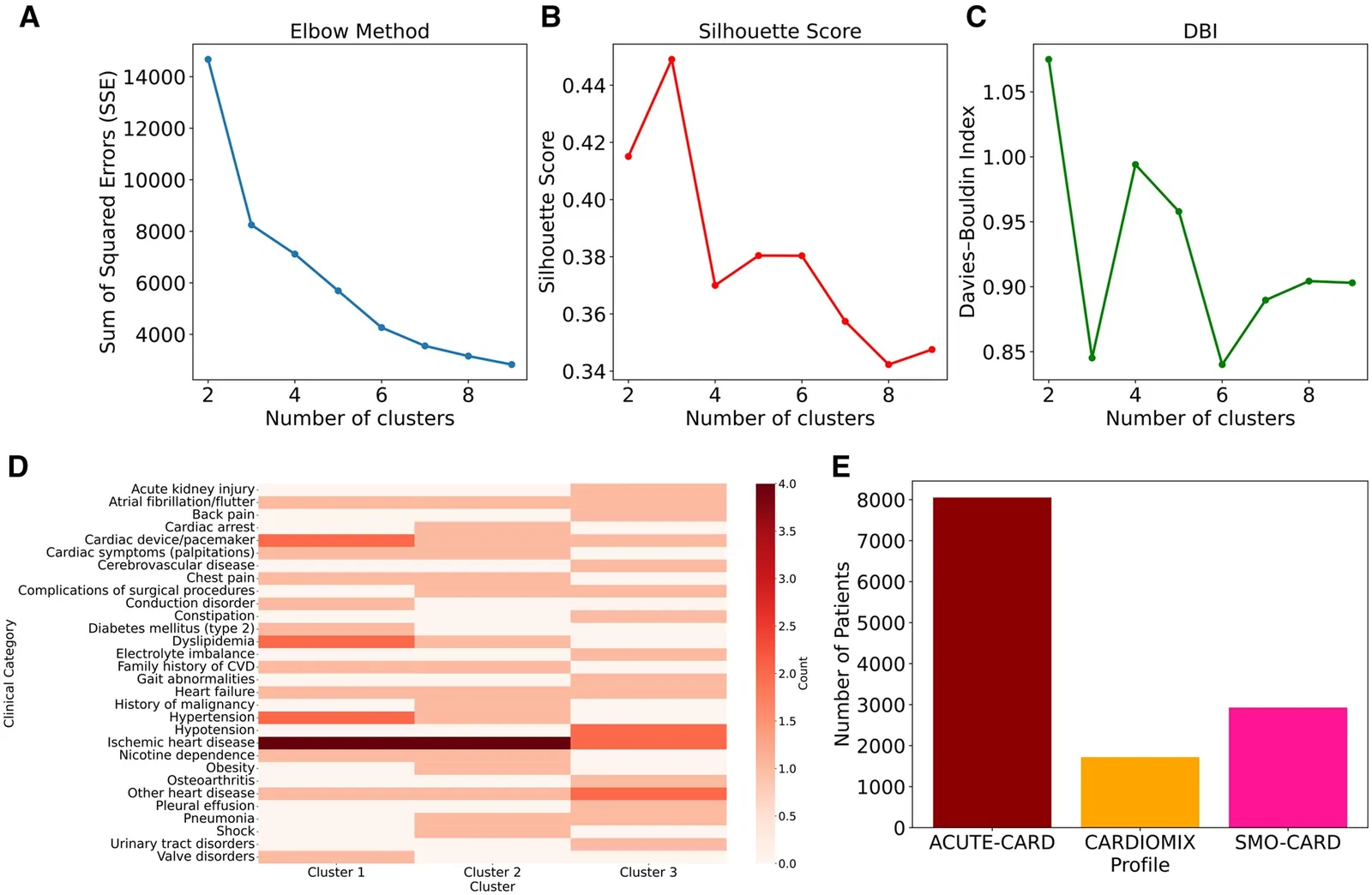
DTW‑derived AMI profiles and diagnostic themes. (A) Elbow plot; (B) silhouette scores; (C) Davies–Bouldin index; (D) heatmap of topic model‑derived ICD categories; (E) profile sizes. Colors: ACUTE‑CARD (dark red), CARDIOMIX (orange), SMO‑CARD (pink).

### Topic modeling and co-occurrence networks

LDA yielded coherent diagnostic themes within each profile (Tables S1, S3, and S4). ACUTE-CARD topics reflected chronic cardiometabolic risk factors and episodic cardiorespiratory failure. CARDIOMIX topics captured structural cardiac and metabolic renal pathology. SMO-CARD topics highlighted respiratory frailty multimorbidity and musculoskeletal decline (Figure 2D).

### Prediction of trajectory profile membership

XGBoost achieved the highest multiclass discrimination (weighted accuracy 78.9%; weighted *F*1 0.782; macro *F*1 0.713; AUC-ROC 0.906, 95% CI, 0.895-0.916), while CatBoost performed comparably (weighted accuracy 77.3%; weighted *F*1 0.780; macro *F*1 0.732; AUC-ROC 0.900, 95% CI, 0.889-0.910; Figure 3). Misclassifications occurred mainly between ACUTE-CARD and CARDIOMIX; SMO-CARD was most distinctly predicted. SHAP analysis showed that pre-AMI respiratory diagnoses and high IMD score drove SMO-CARD predictions; atrial fibrillation, valvular disease, and diabetes drove those for CARDIOMIX; hypertension and dyslipidaemia without respiratory markers were the drivers for ACUTE-CARD (Figure 4A-C). Co-occurrence networks reinforced these distinctions: ACUTE-CARD centered on cardiometabolic hubs; CARDIOMIX showed a tightly coupled cardiorenal-metabolic axis; SMO-CARD displayed a diffuse multisystem network in which COPD and respiratory failure occupied central positions (Figure 4D-F). Older age consistently pushed predictions toward SMO-CARD. As a sensitivity analysis to address potential information leakage, all models were reevaluated using a restricted pre-AMI window of 365 to 7 days before the index event. Model performance declined substantially (XGBoost AUC-ROC 0.635, 95% CI, 0.617-0.652; see Table S2). This reduction reflects the high informativeness of near-AMI diagnoses and shows that excluding the final week leading up to AMI materially diminishes the ability to discriminate between multimorbidity profiles.

**Figure 3.**
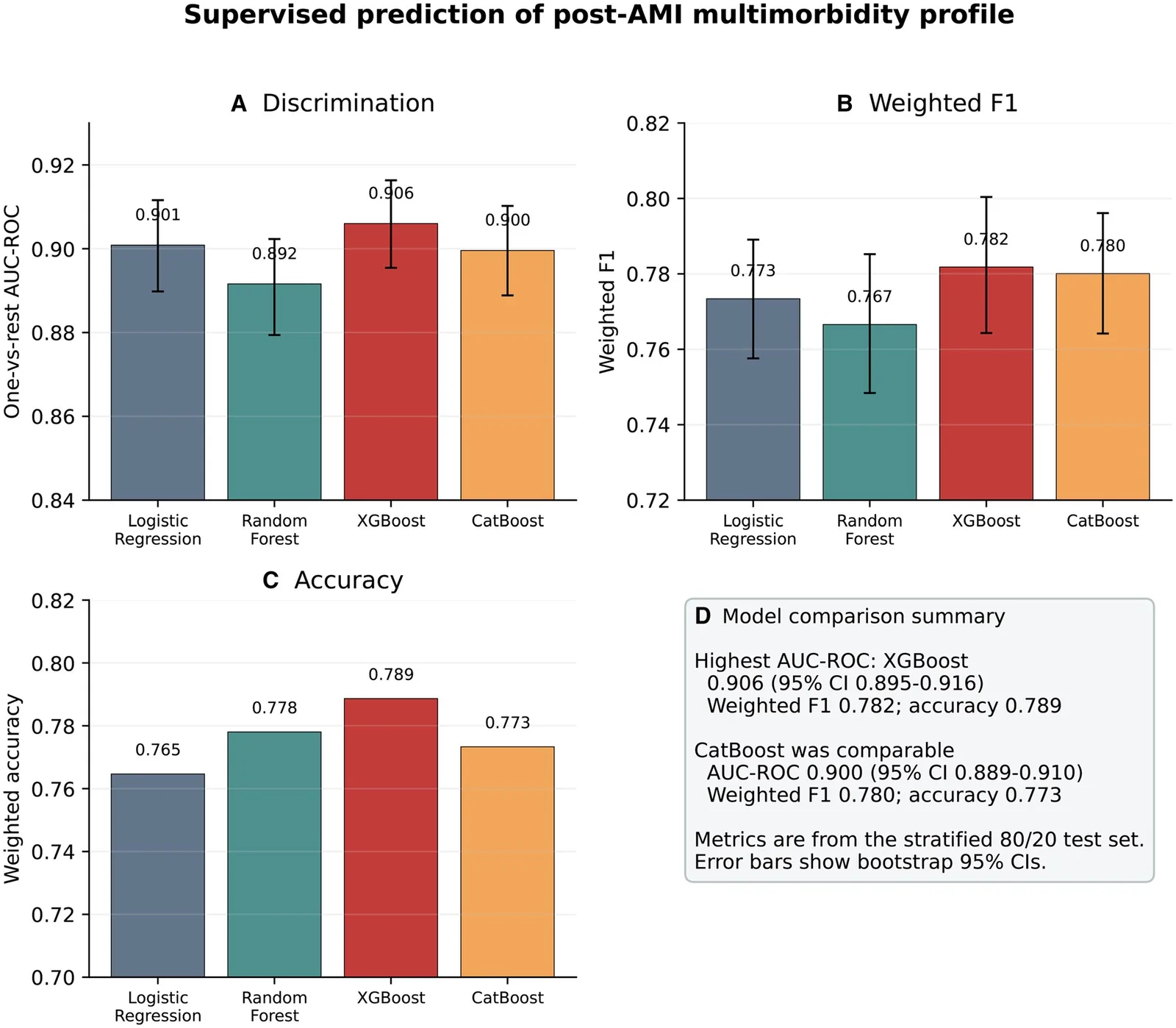
Supervised prediction of post-AMI multimorbidity profile. Panels show test-set model performance for logistic regression, random forest, XGBoost, and CatBoost: (A) one-vs-rest AUC-ROC with bootstrap 95% confidence intervals; (B) weighted *F*1 with bootstrap 95% confidence intervals; (C) weighted accuracy; and (D) summary of the highest-discrimination model and comparable CatBoost performance.

**Figure 4.**
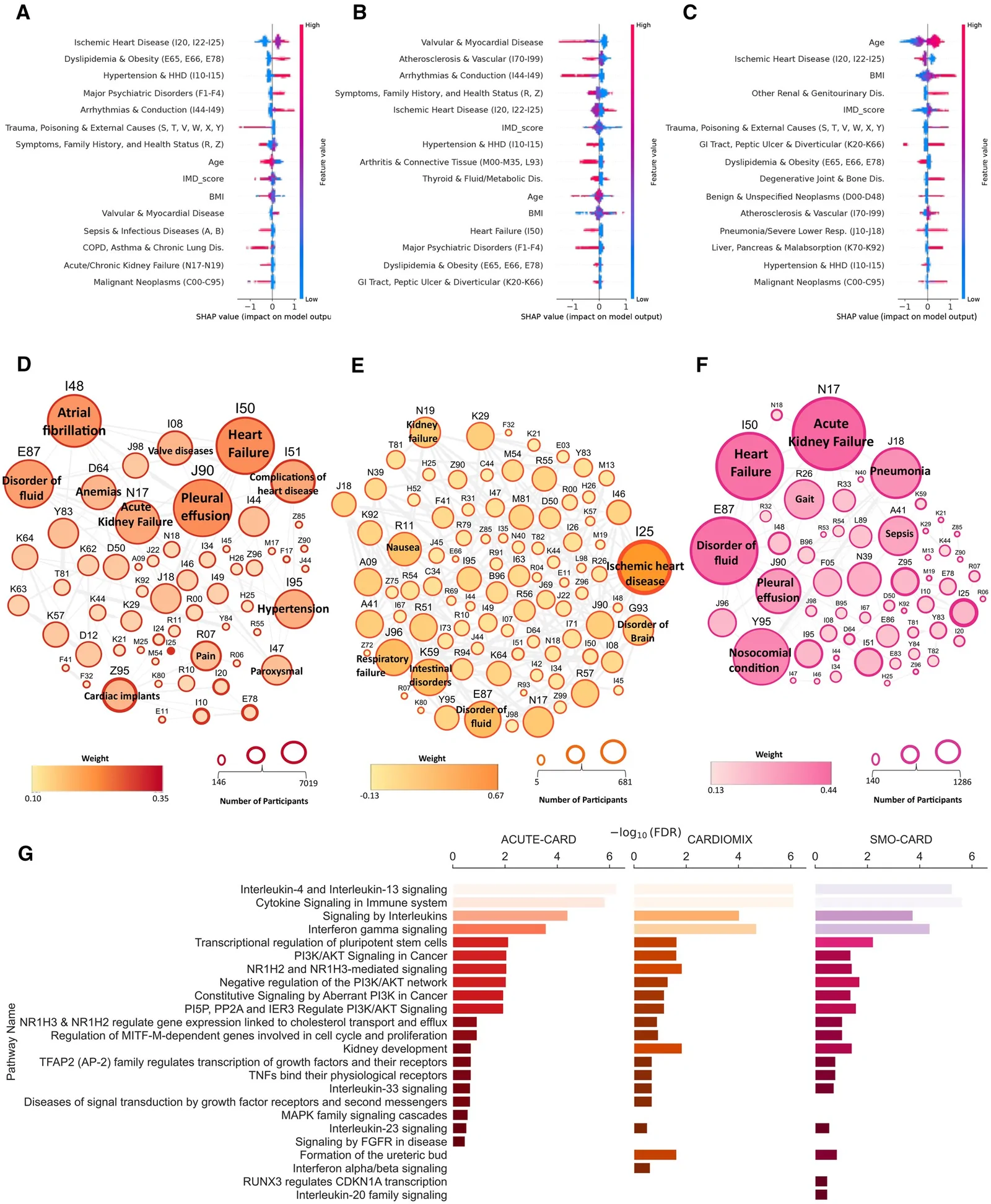
SHAP‑based feature importance, disease co‑occurrence networks, and Reactome pathway enrichment by profile. (A-C) SHAP summary plots (top 20 features). (D-F) Network graphs of top ICD‑10 codes from LDA topics. (G) Top enriched pathways per profile.

### Mortality and SMART

A survival model including trajectory assignment alone showed modest discrimination (C-index 0.59, Model 1) while the model including the SMART score alone resulted in a slightly better C-index of 0.65 (HR per unit 1.05, Model 2). Combining both trajectory assignment and SMART score further improved discrimination marginally (C-index 0.67, Model 3). In the fully adjusted model (Model 4, additionally controlling for age, sex, IMD, SBP, diabetes, and prior vascular disease), the effects of trajectory membership were attenuated to non-significance, consistent with SMART scores capturing the same prognostic variance. Kaplan-Meier curves showed clear unadjusted survival separation with early SMO-CARD divergence (see Figure S1, Tables S5).

### Genetic associations and pathway enrichment

PheWAS identified 2839, 2913, and 2808 significant SNPs for ACUTE-CARD, CARDIOMIX, and SMO-CARD, respectively. Despite a shared coronary artery disease genetic core, pathway enrichment diverged: ACUTE-CARD showed enrichment of innate immune activation, chemokine signaling, and extracellular matrix pathways; CARDIOMIX showed insulin signaling, lipid transport, and atrial conduction pathways consistent with its TCF7L2 association; SMO-CARD showed chronic inflammatory, connective tissue, and smoking-related degenerative pathways. These orthogonal biological signals confirm that DTW-derived clusters capture genuine pathophysiological differences (Figures S2 and S3).

## Discussion

This study applied explainable temporal machine-learning methods to characterise trajectories of multimorbidity after AMI. By encoding post-AMI diagnoses as age-indexed sequences and clustering with DTW, we identified 3 clinically interpretable, biologically grounded trajectories that complement rather than replace gold-standard clinical tools such as the SMART score. Existing comorbidity tools treat multimorbidity as a static state; our approach preserves the ordering and temporal spacing of diagnoses, revealing how disease burden evolves over time. The explicit treatment of time distinguishes ACUTE-CARD (explosive early accumulation) from SMO-CARD (progressive multisystem accrual), even among patients with comparable final diagnosis counts. Explainable supervised learning addresses the well-recognised criticism that machine-learning models function as “black boxes”: SHAP-derived attributions align with clinical reasoning and directly identify actionable pre-AMI predictors.^20^^,^^37–41^

The attenuation of the effects of trajectory assignment in fully adjusted survival models is somewhat expected. The SMART score already encodes age, blood pressure, diabetes, and prior vascular disease—the same factors driving trajectory differences. The trajectories, however, reveal how those risk factors cluster across organ systems and evolve over time. For example, 2 patients with identical SMART scores at AMI incidence may belong to entirely different trajectories. SMO-CARD patients may benefit from pulmonary rehabilitation and geriatric assessment; CARDIOMIX patients from arrhythmia surveillance and nephrology input; ACUTE-CARD patients from cardiometabolic optimisation. These distinctions are not apparent from a risk score alone.^11^^,^^15–18^

Biological characterisation through PheWAS and Reactome enrichment confirms clusters are not statistical artifacts. The ACUTE-CARD profile shows enrichment of inflammatory and matrix-remodeling pathways, while CARDIOMIX of metabolic-electrical pathways anchored to TCF7L2 and the SMO-CARD profile of chronic inflammatory, degenerative, and smoking-related pathways. This approach linking EHR phenotypes to molecular processes exemplifies precision medicine informatics.^28^^,^^29^

### Clinical implications

The pre-AMI classifier demonstrates the potential for such an approach to be embedded in hospital systems to assign provisional trajectory labels at AMI, enabling trajectory-specific care pathways alongside the SMART score. Our sensitivity analysis has demonstrated that diagnoses received in the week leading up to the AMI event have a significant (negative) impact on the classifier’s ability to predict post-AMI trajectory membership. This could potentially be driven by the exclusion of diagnosis codes that are part of more complex AMI presentations recorded in the days leading up to AMI, or possibly even “misdiagnoses” of AMI which may be associated more with one profile over others. Regardless, the approach may still prove useful in clinical settings as it can be used for provisional stratification alongside established clinical risk scores. Nevertheless, validation in diverse populations and prospective implementation studies are required before clinical deployment.

### Limitations and strengths

The UK Biobank exhibits a well-documented healthy participant selection bias: participants are on average healthier, wealthier, and less deprived than the general UK population, leading to lower baseline rates of comorbidity and mortality.^42^ This selection bias may underestimate the true prevalence and complexity of multimorbidity trajectories and the predictive importance of deprivation-related features in real-world clinical populations. However, the UK Biobank’s exceptional scale, linkage to real-world longitudinal health records, and comprehensive genetic data make it uniquely suited for the discovery of multimorbidity trajectory patterns, which are likely to generalise, at least to some extent, across other cohorts. Future work should validate these trajectories and the predictive models in more representative cohorts such as Clinical Practice Research Datalink (CPRD) or the SAIL Databank. The cohort is predominantly White British, limiting generalisability. Diagnoses were derived from hospital ICD-10 codes, missing primary care conditions and sub-annual dynamics. The observational design precludes causal inference. Strengths include the large, deeply phenotyped cohort with long-term follow-up, robust DTW clustering with multi-metric validation, interpretable explainable learning, and integrative genomic validation anchoring EHR phenotypes in biology.

## Conclusion

Explainable temporal modeling of EHR data identifies 3 clinically interpretable, biologically grounded multimorbidity trajectories following AMI that are predictable from pre-AMI data and complement existing clinical risk scores by revealing why risk is high and how it might best be addressed. This application provides a reproducible template for transforming longitudinal EHR data into mechanistically informed phenotypes that support precision care.

## Supplementary Material

ocag135_Supplementary_Data

## Data Availability

Data used in this study were obtained from the UK Biobank resource and cannot be shared directly by the authors under the terms of the UK Biobank Material Transfer Agreement. Access to the underlying individual-level data can be obtained by bona fide researchers through application to UK Biobank (www.ukbiobank.ac.uk) under their standard access procedures (application 83 988). All code used for data processing, trajectory modeling, and prediction, as well as summary-level pathway analysis outputs, are openly available at https://github.com/Donmaston09/AMI_multimorbidity_prediction.
