# Supplementary material for: Explainable temporal machine learning of multimorbidity trajectories after acute myocardial infarction: complementing clinical risk scores with mechanistic phenotypes
Source: J Am Med Inform Assoc. 2026 Aug 6;33(11):2092–101. doi: 10.1093/jamia/ocag135 (PMC13687755; doi:10.1093/jamia/ocag135)
Supplement: Supplementary file 1 [file ocag135_supplementary_data.docx]

## **Supplementary**

### **Supplementary Figure Legends**

**Supplementary Figure 1. Kaplan–Meier curves for composite events, stratified by multimorbidity profiles.**

The Kaplan-Meier of the profiles were considered for All-cause events (within five years). The survival curves are colour-coded for each profile: ACUTE-CARD (dark red), CARDIOMIX (orange), and SMO-CARD (pink).

**Supplementary Figure 2.** **Comparative Pathway Enrichment and Gene Overlap Analysis of AMI-Associated SNPs Across ACUTE-CARD, CARDIOMIX, and SMO-CARD.** Multi-panel figure illustrating gene overlap and pathway enrichment for genes containing acute AMI-associated SNPs in three profiles. **(a)** Top enriched Reactome pathways specific to the ACUTE-CARD, ranked by -log₁₀(FDR) (dark red to orange bars). **(b)** Venn diagram showing counts of overlapping genes across ACUTE-CARD, CARDIOMIX, and SMO-CARD profiles. **(c)** Top enriched Reactome pathways specific to the CARDIOMIX, ranked by -log₁₀(FDR) (yellow-orange bars). **(d)** Top enriched Reactome pathways specific to the SMO-CARD cohort or a complementary gene set, ranked by -log₁₀(FDR) (pink bars).

**Supplementary Figure 3. Statistical significance and effect size of genetic associations from a PheWAS analysis across the profiles**: **(a)** ACUTE-CARD, **(b)** CARDIOMIX, and **(c)** SMO-CARD. X-axis: effect size (log₂OR), Y-axis: significance (−log₁₀p). Dashed lines mark thresholds: log₂OR=±0.5 (vertical) and p=0.05 (horizontal). Red = significant positive associations, blue = significant negative associations, grey = non-significant. Labels show top 10 most significant up and down-regulated genes.

**Code Availability.** The complete computational pipeline and source code for data preprocessing, longitudinal clustering (DTW-k-means), supervised machine learning models (CatBoost, XGBoost), PheWAS, topic modeling (LDA), and network analysis used in this study are publicly available on GitHub at: <https://github.com/Donmaston09/AMI_multimorbidity_prediction>. The repository contains documentation, library dependencies (requirements.txt), and reproducible scripts used to generate the figures and tables presented in the main manuscript.

### **Supplementary Figures**

**Supplementary Figure 1**

**Supplementary Figure 2**

**Supplementary Figure 3**

### **Supplementary Tables**

**Table S1:** Association of Post-AMI Disease-specific Clusters With ICD-10 Themes Derived from Latent Dirichlet Allocation (LDA) Analysis

| **ICD10 code** | **Health condition** | **Theme** | **ACUTE-CARD (count)** | **CARDIOMIX (count)** | **SMO-CARD (count)** | **Total count** | **p-value** | **FDR adj p-value** |
| --- | --- | --- | --- | --- | --- | --- | --- | --- |
| I25 | Chronic ischemic heart disease | Theme 1, Theme 2 | 3472 | 264 | 5250 | 8986 | <0.0001 | <0.0001 |
| Z95 | Presence of cardiac and vascular implants and grafts | Theme 1, Theme 2 | 2534 | 31 | 2258 | 4823 | <0.0001 | <0.0001 |
| I10 | Essential (primary) hypertension | Theme 1, Theme 2 | 1578 | 19 | 2174 | 3771 | <0.0001 | <0.0001 |
| E78 | Disorders of lipoprotein metabolism and other lipidemias | Theme 1, Theme 2 | 1541 | 19 | 2202 | 3762 | <0.0001 | <0.0001 |
| Z82 | Family history of certain disabilities and chronic diseases (leading to disablement) | Theme 1, Theme 2 | 1116 | 24 | 1982 | 3122 | <0.0001 | <0.0001 |
| I20 | Angina pectoris | Theme 1, Theme 2 | 1364 | 11 | 1380 | 2755 | <0.0001 | <0.0001 |
| I51 | Complications and ill-defined descriptions of heart disease | Theme 1, Theme 2 | 1528 | 25 | 1061 | 2614 | <0.0001 | <0.0001 |
| I50 | Heart failure | Theme 1, Theme 2 | 1657 | 27 | 803 | 2487 | <0.0001 | <0.0001 |
| R07 | Pain in throat and chest | Theme 1, Theme 2 | 1093 | 7 | 840 | 1940 | <0.0001 | <0.0001 |
| I48 | Atrial fibrillation and flutter | Theme 1, Theme 2 | 1250 | 15 | 535 | 1800 | <0.0001 | <0.0001 |
| I24 | Other acute ischemic heart diseases | Theme 1, Theme 2 | 1006 | 12 | 709 | 1727 | <0.0001 | <0.0001 |
| N17 | Acute kidney failure | Theme 1, Theme 2 | 1283 | 8 | 151 | 1442 | <0.0001 | <0.0001 |
| E87 | Other disorders of fluid, electrolyte and acid-base balance | Theme 1, Theme 2 | 1156 | 7 | 201 | 1364 | <0.0001 | <0.0001 |
| I08 | Multiple valve diseases | Theme 1, Theme 2 | 841 | 7 | 510 | 1358 | <0.0001 | <0.0001 |
| I95 | Hypotension | Theme 1, Theme 2 | 1044 | 7 | 276 | 1327 | <0.0001 | <0.0001 |
| E66 | Overweight and obesity | Theme 1, Theme 2 | 719 | 9 | 542 | 1270 | <0.0001 | <0.0001 |
| I44 | Atrioventricular and left bundle-branch block | Theme 1, Theme 2 | 774 | 11 | 385 | 1170 | <0.0001 | <0.0001 |
| J18 | Pneumonia, unspecified organism | Theme 1, Theme 2 | 988 | 12 | 150 | 1150 | <0.0001 | <0.0001 |
| R00 | Abnormalities of heartbeat | Theme 1, Theme 2 | 697 | 7 | 383 | 1087 | <0.0001 | <0.0001 |
| F17 | Nicotine dependence | Theme 2 | 450 | 6 | 612 | 1068 | <0.0001 | <0.0001 |
| N18 | Chronic kidney disease (CKD) | Theme 1, Theme 2 | 808 | 7 | 217 | 1032 | <0.0001 | <0.0001 |
| Y83 | Surgical operation and other surgical procedures as the cause of abnormal reaction of the patient, or of later complication, without mention of misadventure at the time of the procedure | Theme 1, Theme 2 | 808 | 5 | 182 | 995 | <0.0001 | <0.0001 |
| D64 | Other anemias | Theme 1, Theme 2 | 862 | 4 | 129 | 995 | <0.0001 | <0.0001 |
| K21 | Gastro-esophageal reflux disease | Theme 1, Theme 2 | 629 | 3 | 361 | 993 | <0.0001 | <0.0001 |
| J90 | Pleural effusion, not elsewhere classified | Theme 1, Theme 2 | 848 | 8 | 119 | 975 | <0.0001 | <0.0001 |

Acute Cardiorenal–Respiratory with Chronic Metabolic Disease (ACUTE-CARD), Cardiometabolic with Mixed Arrhythmic–Ischaemic Burden (CARDIOMIX), Smoking-Related Cardiovascular Disease with Multimorbidity (SMO-CARD). p-value: The statistical significance of the difference in prevalence of the health condition across the three profiles. FDR adj p-value: The p-value adjusted for multiple testing using the False Discovery Rate (FDR) method. This adjustment helps to reduce the chance of false positives. Themes were derived from an LDA topic-modelling analysis of ICD-10 codes to identify clinically coherent clusters of comorbidities.

**Table S2. Supervised model performance in primary and 7-day sensitivity windows**

| **Window** | **Model** | **Accuracy** | **Weighted F1** | **Weighted F1 95% CI** | **Macro F1** | **AUC-ROC** | **AUC-ROC 95% CI** | **p-value** |
| --- | --- | --- | --- | --- | --- | --- | --- | --- |
| Primary pre-AMI window: 365 to 1 day before AMI | Logistic Regression | 0.765 | 0.773 | 0.758-0.789 | 0.729 | 0.901 | 0.890-0.912 | <0.001 |
| Primary pre-AMI window: 365 to 1 day before AMI | Random Forest | 0.778 | 0.767 | 0.748-0.785 | 0.698 | 0.892 | 0.879-0.902 | <0.001 |
| Primary pre-AMI window: 365 to 1 day before AMI | XGBoost | 0.789 | 0.782 | 0.764-0.800 | 0.713 | 0.906 | 0.895-0.916 | <0.001 |
| Primary pre-AMI window: 365 to 1 day before AMI | CatBoost | 0.773 | 0.780 | 0.764-0.796 | 0.732 | 0.900 | 0.889-0.910 | <0.001 |
| Sensitivity pre-AMI window: 365 to 7 days before AMI | Logistic Regression | 0.217 | 0.156 | 0.141-0.172 | 0.231 | 0.639 | 0.622-0.656 | <0.001 |
| Sensitivity pre-AMI window: 365 to 7 days before AMI | Random Forest | 0.640 | 0.575 | 0.553-0.598 | 0.384 | 0.619 | 0.601-0.639 | <0.001 |
| Sensitivity pre-AMI window: 365 to 7 days before AMI | XGBoost | 0.654 | 0.578 | 0.555-0.600 | 0.377 | 0.635 | 0.617-0.652 | <0.001 |
| Sensitivity pre-AMI window: 365 to 7 days before AMI | CatBoost | 0.400 | 0.433 | 0.415-0.452 | 0.374 | 0.622 | 0.603-0.641 | <0.001 |

Note: Weighted average scores were used to evaluate the multiclass performance. Performance metrics are reported for the stratified 80/20 test set. Confidence intervals were estimated using 1,000 bootstrap resamples. Statistical significance was inferred from whether the 95% confidence interval excluded the null value of 0.5.

**Table S3:** Key Health Conditions Across Multimorbidity Profiles in pre-AMI data

| **ICD10 code** | **Health condition** | **ACUTE-CARD (count)** | **CARDIOMIX (count)** | **SMO-CARD (count)** | **ACUTE-CARD prevalence** | **CARDIOMIX prevalence** | **SMO-CARD prevalence** | **Total count** | **p-value** | **FDR adj**  **p-value** |
| --- | --- | --- | --- | --- | --- | --- | --- | --- | --- | --- |
| I25 | Chronic ischemic heart disease | 2371 | 277 | 4037 | 0.45 | 0.30 | 0.62 | 6685 | <0.0001 | <0.0001 |
| E78 | Disorders of lipoprotein metabolism and other lipidemias | 1037 | 54 | 1803 | 0.2 | 0.06 | 0.28 | 2894 | <0.0001 | <0.0001 |
| I10 | Essential (primary) hypertension | 1001 | 67 | 1721 | 0.19 | 0.07 | 0.27 | 2789 | <0.0001 | <0.0001 |
| Z82 | Family history of certain disabilities and chronic diseases (leading to disablement) | 838 | 52 | 1710 | 0.16 | 0.06 | 0.26 | 2600 | <0.0001 | <0.0001 |
| I51 | Complications and ill-defined descriptions of heart disease | 884 | 58 | 855 | 0.17 | 0.06 | 0.13 | 1797 | <0.0001 | <0.0001 |
| I50 | Heart failure | 934 | 60 | 650 | 0.18 | 0.07 | 0.1 | 1644 | <0.0001 | <0.0001 |
| I20 | Angina pectoris | 628 | 47 | 706 | 0.12 | 0.05 | 0.11 | 1381 | <0.0001 | <0.0001 |
| I48 | Atrial fibrillation and flutter | 671 | 51 | 399 | 0.13 | 0.06 | 0.06 | 1121 | <0.0001 | <0.0001 |
| Z95 | Presence of cardiac and vascular implants and grafts | 436 | 49 | 571 | 0.08 | 0.05 | 0.09 | 1056 | 0.0015 | 0.0078 |
| F17 | Nicotine dependence | 405 | 18 | 601 | 0.08 | 0.02 | 0.09 | 1024 | <0.0001 | <0.0001 |
| I08 | Multiple valve diseases | 504 | 17 | 423 | 0.1 | 0.02 | 0.07 | 944 | <0.0001 | <0.0001 |
| I44 | Atrioventricular and left bundle-branch block | 474 | 29 | 342 | 0.09 | 0.03 | 0.05 | 845 | <0.0001 | <0.0001 |
| N17 | Acute kidney failure | 596 | 38 | 170 | 0.11 | 0.04 | 0.03 | 804 | <0.0001 | <0.0001 |
| E11 | Type 2 diabetes mellitus | 373 | 32 | 378 | 0.07 | 0.03 | 0.06 | 783 | <0.0001 | 0.0003 |
| R07 | Pain in throat and chest | 368 | 47 | 366 | 0.07 | 0.05 | 0.06 | 781 | 0.005 | 0.0204 |
| E66 | Overweight and obesity | 349 | 22 | 402 | 0.07 | 0.02 | 0.06 | 773 | <0.0001 | <0.0001 |
| I24 | Other acute ischemic heart diseases | 356 | 45 | 352 | 0.07 | 0.05 | 0.05 | 753 | 0.0045 | 0.0187 |
| E87 | Other disorders of fluid, electrolyte and acid-base balance | 502 | 31 | 201 | 0.09 | 0.03 | 0.03 | 734 | <0.0001 | <0.0001 |
| J18 | Pneumonia, unspecified organism | 499 | 47 | 139 | 0.09 | 0.05 | 0.02 | 685 | <0.0001 | <0.0001 |
| I46 | Cardiac arrest | 274 | 39 | 326 | 0.05 | 0.04 | 0.05 | 639 | 0.4779 | 0.6429 |

Acute Cardiorenal–Respiratory with Chronic Metabolic Disease (ACUTE-CARD), Cardiometabolic with Mixed Arrhythmic–Ischaemic Burden (CARDIOMIX), Smoking-Related Cardiovascular Disease with Multimorbidity (SMO-CARD). Disease prevalence (%): The percentage of participants within each profile who have the specified health condition, p-value: The statistical significance of the difference in prevalence of the health condition across the three profiles. FDR adj p-value: The p-value adjusted for multiple testing using the False Discovery Rate (FDR) method. This adjustment helps to reduce the chance of false positives.

**Table S4:** Key Health Conditions Across Multimorbidity Profiles in post-AMI data

| **ICD10 code** | **Health condition** | **ACUTE-CARD (count)** | **CARDIOMIX (count)** | **SMO-CARD (count)** | **ACUTE-CARD prevalence** | **CARDIOMIX prevalence** | **SMO-CARD prevalence** | **Total count** | **p-value** | **FDR adj p-value** |
| --- | --- | --- | --- | --- | --- | --- | --- | --- | --- | --- |
| I25 | Chronic ischemic heart disease | 3472 | 264 | 5250 | 0.66 | 0.29 | 0.81 | 8986 | <0.0001 | <0.0001 |
| Z95 | Presence of cardiac and vascular implants and grafts | 2534 | 31 | 2258 | 0.48 | 0.03 | 0.35 | 4823 | <0.0001 | <0.0001 |
| I10 | Essential (primary) hypertension | 1578 | 19 | 2174 | 0.3 | 0.02 | 0.34 | 3771 | <0.0001 | <0.0001 |
| E78 | Disorders of lipoprotein metabolism and other lipidemias | 1541 | 19 | 2202 | 0.29 | 0.02 | 0.34 | 3762 | <0.0001 | <0.0001 |
| Z82 | Family history of certain disabilities and chronic diseases (leading to disablement) | 1116 | 24 | 1982 | 0.21 | 0.03 | 0.31 | 3122 | <0.0001 | <0.0001 |
| I20 | Angina pectoris | 1364 | 11 | 1380 | 0.26 | 0.01 | 0.21 | 2755 | <0.0001 | <0.0001 |
| I51 | Complications and ill-defined descriptions of heart disease | 1528 | 25 | 1061 | 0.29 | 0.03 | 0.16 | 2614 | <0.0001 | <0.0001 |
| I50 | Heart failure | 1657 | 27 | 803 | 0.31 | 0.03 | 0.12 | 2487 | <0.0001 | <0.0001 |
| R07 | Pain in throat and chest | 1093 | 7 | 840 | 0.21 | 0.01 | 0.13 | 1940 | <0.0001 | <0.0001 |
| I48 | Atrial fibrillation and flutter | 1250 | 15 | 535 | 0.24 | 0.02 | 0.08 | 1800 | <0.0001 | <0.0001 |
| I24 | Other acute ischemic heart diseases | 1006 | 12 | 709 | 0.19 | 0.01 | 0.11 | 1727 | <0.0001 | <0.0001 |
| N17 | Acute kidney failure | 1283 | 8 | 151 | 0.24 | 0.01 | 0.02 | 1442 | <0.0001 | <0.0001 |
| E87 | Other disorders of fluid, electrolyte and acid-base balance | 1156 | 7 | 201 | 0.22 | 0.01 | 0.03 | 1364 | <0.0001 | <0.0001 |
| I08 | Multiple valve diseases | 841 | 7 | 510 | 0.16 | 0.01 | 0.08 | 1358 | <0.0001 | <0.0001 |
| I95 | Hypotension | 1044 | 7 | 276 | 0.2 | 0.01 | 0.04 | 1327 | <0.0001 | <0.0001 |
| E66 | Overweight and obesity | 719 | 9 | 542 | 0.14 | 0.01 | 0.08 | 1270 | <0.0001 | <0.0001 |
| I44 | Atrioventricular and left bundle-branch block | 774 | 11 | 385 | 0.15 | 0.01 | 0.06 | 1170 | <0.0001 | <0.0001 |
| J18 | Pneumonia, unspecified organism | 988 | 12 | 150 | 0.19 | 0.01 | 0.02 | 1150 | <0.0001 | <0.0001 |
| R00 | Abnormalities of heartbeat | 697 | 7 | 383 | 0.13 | 0.01 | 0.06 | 1087 | <0.0001 | <0.0001 |
| F17 | Nicotine dependence | 450 | 6 | 612 | 0.09 | 0.01 | 0.09 | 1068 | <0.0001 | <0.0001 |

Acute Cardiorenal–Respiratory with Chronic Metabolic Disease (ACUTE-CARD), Cardiometabolic with Mixed Arrhythmic–Ischaemic Burden (CARDIOMIX), Smoking-Related Cardiovascular Disease with Multimorbidity (SMO-CARD). Disease prevalence (%): The percentage of participants within each profile who have the specified health condition, p-value: The statistical significance of the difference in prevalence of the health condition across the three profiles. FDR adj p-value: The p-value adjusted for multiple testing using the False Discovery Rate (FDR) method. This adjustment helps to reduce the chance of false positives.

**Table S5**: Cox proportional hazards models (Models 1–4) for 0–5-year all-cause mortality

| **Model** | **Covariate** | **HR (95% CI)** | **p-value** |
| --- | --- | --- | --- |
| 1. Profiles only | Profile (per unit increase) | 1.40 (1.23 – 1.59) | <0.001 |
| 2. SMART-risk only | SMART-risk score (per unit) | 1.05 (1.04 – 1.07) | <0.001 |
| 3. Profiles + SMART-risk | Profile (per unit increase) | 1.25 (1.10 – 1.43) | 0.001 |
|  | SMART-risk score (per unit) | 1.05 (1.04 – 1.06) | <0.001 |
| 4. Fully adjusted (Profile + SMART-risk + covariates) | Profile (per unit increase) | 1.12 (0.98 – 1.29) | 0.105 |
|  | SMART-risk score | 1.03 (1.02 – 1.05) | <0.001 |
|  | Age at baseline | 1.06 (1.03 – 1.08) | <0.001 |
|  | Sex (male) | 0.71 (0.54 – 0.95) | 0.019 |
|  | IMD score | 1.01 (1.00 – 1.02) | 0.002 |
|  | BMI | 1.00 (0.97 – 1.02) | 0.739 |
|  | Diabetes | 1.50 (1.04 – 2.17) | 0.031 |
|  | CAD | 1.21 (0.84 – 1.72) | 0.302 |
|  | Cerebrovascular disease | 1.06 (0.53 – 2.15) | 0.862 |
|  | PAD | 1.17 (0.61 – 2.23) | 0.638 |

Values are hazard ratios (HR) with 95% confidence intervals. Reference profile = ACUTE-CARD. Model 4 additionally adjusts for all listed covariates.
